# The Roles of Mitophagy and Autophagy in Ineffective Erythropoiesis in β-Thalassemia

**DOI:** 10.3390/ijms231810811

**Published:** 2022-09-16

**Authors:** Pornthip Chaichompoo, Saovaros Svasti, Duncan R. Smith

**Affiliations:** 1 Department of Pathobiology, Faculty of Science, Mahidol University, Bangkok 10400, Thailand; 2 Thalassemia Research Center, Institute of Molecular Biosciences, Mahidol University, Nakhon Pathom 73170, Thailand or; 3 Department of Biochemistry, Faculty of Science, Mahidol University, Bangkok 10400, Thailand; 4 Pathology Laboratory, Institute of Molecular Biosciences, Mahidol University, Nakhon Pathom 73170, Thailand

**Keywords:** autophagy, apoptosis, ER stress, β-thalassemia, ineffective erythropoiesis

## Abstract

β-Thalassemia is one of the most common genetically inherited disorders worldwide, and it is characterized by defective β-globin chain synthesis leading to reduced or absent β-globin chains. The excess α-globin chains are the key factor leading to the death of differentiating erythroblasts in a process termed ineffective erythropoiesis, leading to anemia and associated complications in patients. The mechanism of ineffective erythropoiesis in β-thalassemia is complex and not fully understood. Autophagy is primarily known as a cell recycling mechanism in which old or dysfunctional proteins and organelles are digested to allow recycling of constituent elements. In late stage, erythropoiesis autophagy is involved in the removal of mitochondria as part of terminal differentiation. Several studies have shown that autophagy is increased in earlier erythropoiesis in β-thalassemia erythroblasts, as compared to normal erythroblasts. This review summarizes what is known about the role of autophagy in β-thalassemia erythropoiesis and shows that modulation of autophagy and its interplay with apoptosis may provide a new therapeutic route in the treatment of β-thalassemia. Literature was searched and relevant articles were collected from databases, including PubMed, Scopus, Prospero, Clinicaltrials.gov, Google Scholar, and the Google search engine. Search terms included: β-thalassemia, ineffective erythropoiesis, autophagy, novel treatment, and drugs during the initial search. Relevant titles and abstracts were screened to choose relevant articles. Further, selected full-text articles were retrieved, and then, relevant cross-references were scanned to collect further information for the present review.

## 1. Introduction

β-Thalassemia, one of the most common genetic inheritance disorders worldwide, is caused by the absence or reduced presence of β-globin chains in hemoglobin (Hb) molecules [1]. The Hb-producing cells accumulate excess α-globin, which forms cytotoxic intracellular precipitates that impair erythroid cell production in a process known as ineffective erythropoiesis, a major determinant of β-thalassemia pathophysiology [2]. Erythrokinetic and ferrokinetic studies in β-thalassemia patients indicated that about 65% of erythroblasts die before becoming mature red blood cells (RBCs) [3]. Autophagy plays an important role in organelle clearance during terminal erythroid differentiation of erythrocytes [4], in addition to its cellular role of recycling macromolecules in cells to maintain homeostasis [5]. Autophagy can be induced by extracellular or intracellular stresses such as nutrient and growth factor deprivation [6], hypoxia [7], calcium (Ca^2+^) dysregulation [8], endoplasmic reticulum (ER) stress [9], and oxidative stress [10]. Herein, the role of autophagy in β-thalassemic erythropoiesis is reviewed.

## 2. Clinical Manifestation of β-Thalassemia and β-Thalassemia/HbE Diseases

### 2.1. Etiology and Epidemiology of β-Thalassemia and β-Thalassemia/HbE Diseases

The Hb tetramer consists of two different pairs of globin chains and heme, such as adult Hb or HbA which consists of two α-globin and two β-globin chains (α_2_β_2_) together with four heme-iron complexes. It is Hb that provides RBCs with the capacity to carry oxygen molecules to tissues and to remove carbon dioxide. Hemoglobinopathy is characterized by a reduction in globin production (called thalassemia) or by the presence of an abnormal Hb, such as HbS (Sickle cell hemoglobin; α_2_β^S^_2_), which results from a single base mutation of the β-globin gene at codon 6, leading to an amino acid change from glutamine to valine [11]. Additionally, HbE (α_2_β^E^_2_), the second most prevalent abnormal Hb of the world, is both a thalassemia and an abnormal Hb. In HbE, there is a single base mutation of β-globin gene at codon 26 leading to the amino acid change from glutamine to lysine, which additionally creates an aberrant splicing of the β^E^-globin pre-mRNA leading to a reduction of β^E^-globin chains [12].

**Figure 1:**
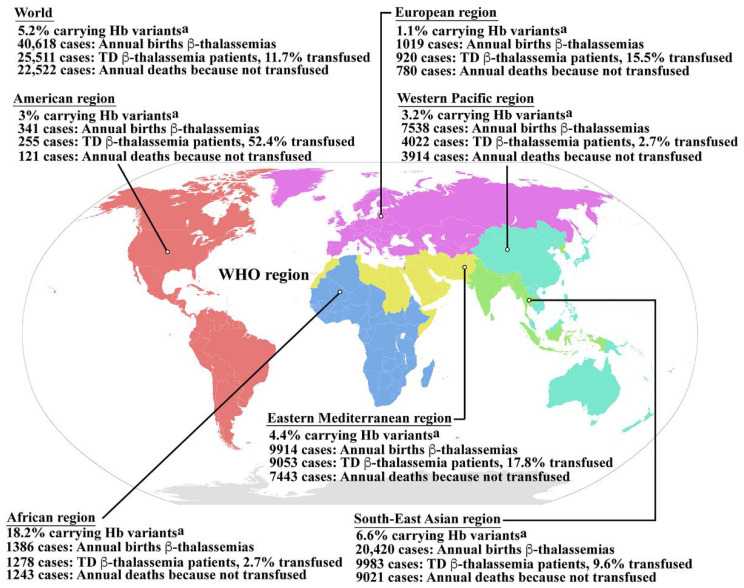
Epidemiology of β-thalassemia. Global epidemiological data including the percentages of populations carrying Hb variants, the number of newborns who have β-thalassemias per year, the number of patients who have TDT, and the percentage of TDT patients who are reached for blood transfusion, and the number of patients who died as a consequence of not receiving blood transfusion worldwide and in the individual sub-regions, according to the World Health Organization (WHO) as reported by Modell B and Darlison M published in the Bull World Health Organ 2008, 86, (6), 480–487 [13]. ^a^ Hemoglobin variants including HbS, HbC, HbE, HbD, etc., β-thalassemia and α-thalassemia. Hb; hemoglobin, TD β-thalassemia; transfusion dependent β-thalassemia. Source of WHO region map; wikipedia.org (accessed in September 2022). The map is in the public domain.

Hemoglobinopathy is among the most common genetic disorders worldwide, and can be found in the Mediterranean region, the Middle East, the Indian subcontinent, and throughout Southeast Asia in a line stretching from southern China down the Malaysian peninsula to the Indonesian islands. Hemoglobinopathy is widely distributed, and approximately 5.2% of the world’s population carry significant globin variants including HbS, HbC, HbE, HbD, β-thalassemia, and α-thalassemia (Figure 1) [13]. Therefore, the worldwide birth rate of newborns who are homozygous or compound heterozygous for symptomatic thalassemia, including HbBart’s hydrops fetalis (homozygous α^0^-thalassemia), HbH disease (α^0^/α^+^-thalassemia), β-thalassemia major (homozygous β^0^-thalassemia), and β-thalassemia/HbE disease, is 0.46 per 1000 births. Although the most severe form of thalassemia is HbBart’s hydrops fetalis, the affected fetus dies in utero, and therefore the majority of severe thalassemia patients worldwide are homogygous β-thalassemia and β-thalassemia/HbE. The estimated number of births with β-thalassemia annually is approximately 40,618 of which 25,511 (62.8%) have severe anemia and require blood transfusions. However, only 11.7% of patients requiring blood transfusion can reached for treatment (Figure 1) [13]. In addition, HbE is common in Southeast Asia, Bangladesh, and Northeast India [14]. Increased global migration is a major factor that has increased the prevalence of hemoglobinopathies worldwide, leading to it becoming a global, rather than regional public health problem.

β-Thalassemia is a heterogeneous group of inherited disorders of Hb synthesis that is characterized by the absence or reduced presence of β-globin chains. Over 300 mutations in the β-globin gene that cause the β-thalassemia have been documented [15]. Clinical manifestations range from the β-thalassemia trait, which presents as a borderline asymptomatic anemia (β/β^+^ or β/β^0^); to β-thalassemia intermedia, alternatively termed non-transfusion dependent thalassemia (NTDT), which presents with mild-to-moderate symptoms as a result of a relative reduction in β-globin chain production (β^+^/β^+^ or β^+^/β^0^); to β-thalassemia major, alternatively termed transfusion dependent thalassemia (TDT), which results in severe symptoms as a consequence of the complete absence of β-globin chain synthesis (β^0^/β^0^) [2,16]. The compound heterozygosity caused by co-inheritance of β-thalassemia and HbE, which causes β-thalassemia/HbE disease (β^0^/β^E^ or β^+^/β^E^), can result in a wide range of disease severity, with Hb levels ranging from 3 to 13 g/dL, resulting in mild or moderate (like β-thalassemia intermedia) to severe (like β-thalassemia major) presentation [2,17].

### 2.2. Pathophysiology of β-Thalassemia and β-Thalassemia/HbE Diseases

**Figure 2:**
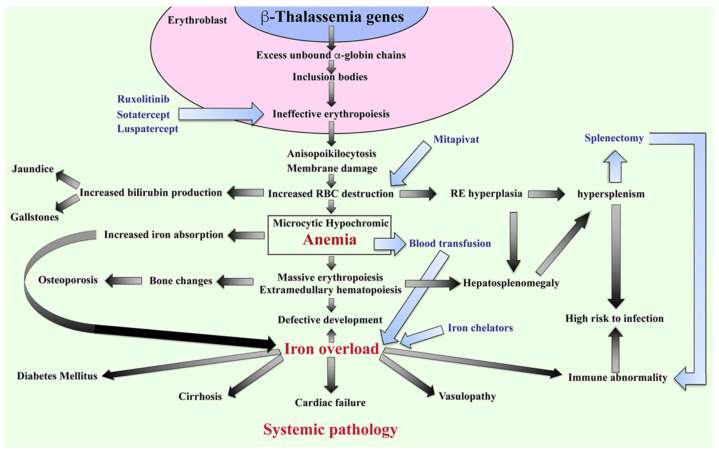
Pathophysiology of β-thalassemia. Defect in β-globin genes leads to reduced or absent β-globin production and consequent excess unbound α-globin chain accumulation and precipitation in erythroblasts, resulting in ineffective eythropoiesis. Abnormal RBCs, including anisopoikilocytosis, and RBCs with damaged membranes cause RBC destruction in the spleen leading to anemia, reticuloendothelial (RE) hyperplasia, and increased bilirubin production. Iron overload is a key factor in inducing a systemic pathology leading to increased mortality and morbidity in β-thalassemia patients. Blood transfusion and iron chelators are the conventional treatments for β-thalassemia. Curently, therapeutic drugs such as ruxolitinib, sotatercept, luspatercept, and mitapivat that target ineffective erythropoiesis by increasing Hb production are in clinical trials. Hb; hemoglobin, RBCs; red blood cells, and RE system; reticuloendothelial system.

The β-thalassemia or β-thalassemia/HbE patients have imbalanced globin chains, with excess unbound α-globin chains in erythroblasts resulting in ineffective erythropoiesis (Figure 2). The patients suffer from chronic anemia that induces erythropoietin production and consequently stimulates massive erythropoiesis, extramedullary hematopoiesis, and increased iron absorption at the gastrointestinal tract [18]. Abnormal thalassemic RBCs are commonly cleared by macrophages in the sinusoids of spleen and by the reticuloendothelial (RE) system, where heme is degraded into a porphyrin ring and iron. The iron is recycled, while the porphyrin ring is oxidized to biliverdin and subsequently converted to unconjugated bilirubin and then passively taken into hepatocytes. High levels of bilirubin can cause jaundice and gallstones in patients. The increased number of damaged RBCs stimulate RE hyperplasia and can induce hepatosplenomegaly. In turn, the increased number of RBCs destroyed in the spleen can trigger hyperfunction of macrophages leading to hypersplenism, resulting in severe anemia. Splenectomy is a conventional treatment to cure hypersplenism, but it subsequently increases susceptibility to infection and can promote thrombosis. In addition, massive erythropoiesis in the bone marrow can induce bone changes that contribute to osteoporosis, while extramedullary hematopoiesis can interfere with organ functions leading to additional complications. The conventional treatment for patients with severe anemia is regular blood transfusions. However, increased gastrointestinal iron absorption together with multiple blood transfusion leads patients to suffer from iron overload, which is a serious complication that plays an important role in induced systemic abnormalities such as cardiovasculopathy, cirrhosis, diabetes mellitus, abnormal immunity, and other complications [19,20,21,22,23,24].

### 2.3. Current Treatment for β-Thalassemia and β-Thalassemia/HbE Diseases

Currently, the recommended guidelines for medical management focus on blood transfusion and iron chelation to delay pathophysiological changes in patients. Therapeutic options such as stimulation of HbF induction using hydroxyurea, which aims to reduce the frequency of blood transfusion, are widely used. Curative treatments such as hematopoietic stem cell transplantation (HSCT) and gene therapy have been established but remain severely limited in application. Therefore, the clinical management for β-thalassemia depends on the age of the patients, the severity of anemia, and the individual responsiveness to treatments.

Blood transfusion: TDT patients who have anemia with Hb levels <7 g/dL require regular blood transfusions to maintain Hb levels of around 9–10 g/dL [25]. However, multiple blood transfusions cause iron overload and have a high risk of alloimmunization as well as an increased risk from blood-borne infectious diseases. Leukocyte-depleted RBCs are recommended to reduce the incidence of febrile and urticarial reactions as well as infectious cytomegalovirus contamination.

Iron chelation: Iron chelation therapy is recommended for patients who have serum ferritin levels of >1000 ng/mL to maintain serum ferritin levels of between 500–1000 ng/mL. The available iron chelators are deferoxamine, deferiprone and deferasirox. Deferoxamine is administered at a dose of 40–50 mg/kg by 8–12 h of subcutaneous infusion 5 days a week. Deferoxamine has the benefit of chelating iron from hepatocytes and cardiomyocytes; however, there is a high risk of Yersinia infections, hearing loss, retinopathy, poor growth, and allergy [26]. Deferiprone has oral tablet and syrup formulations, which are usually administered at 75–100 mg/kg/day. Deferiprone promotes the excretion of storage iron from parenchymal iron stores but has no advantage over deferoxamine in promoting reticuloendothelial iron excretion. The adverse effects of deferiprone are nausea, vomiting, abdominal pain, increased alanine aminotransferase levels, arthralgia, and neutropenia [26]. Combined therapy of deferoxamine and deferiprone has been recommended to treat patients with severe myocardial iron overload and for prevention and/or reversal of endocrinopathies. Deferasirox has dispersible tablet and film-coated tablet formulations that are administered once-daily at a dose of 20 or 30 mg/kg. Deferasirox is recommended for cardiac iron clearance, but even liver iron is lowered. The adverse effects of deferasirox are diarrhea, vomiting, nausea, abdominal pain, rash, increased alanine aminotransferase levels, and increased serum creatinine [26]. The iron chelator dosing and regimen for appropriate management of iron overload to avoid further organ toxicity and preserve organ function are the key concepts of treatment. Therefore, the optimal iron chelation therapy including the initiation time for treatment, close monitoring, and continuous adjustment needs careful consideration.

Stimulation of HbF production: Nowadays, hydroxyurea at a low dose (5–20 mg/kg/day for 5 days a week) is a therapeutic option for β-thalassemia, which aims to reduce the frequency of blood transfusions. However, hydroxyurea has potential severe adverse effects on neutropenia and thrombocytopenia that need careful monitoring. In addition, partial responders and non-responders to hydroxyurea treatment have been reported [27].

Stem cell transplantation: Allogenic HSCT could replace the ineffective endogenous erythropoiesis and can cure the disease. Hematopoietic stem cells (HSCs) can be harvested from peripheral blood and bone marrow of a human leukocyte antigen (HLA)-matched unrelated donor or HLA-haploidentical donor. HLA-identical sibling umbilical cord blood stem cell transplantation (UCBT) is a potential source of HSCs; however, it is limited to the yield of stem cells from a single unit of umbilical cord blood (UCB). Therefore, age and body weight of patient, ex vivo expansion of UCB stem cells, and enhanced homing to the bone marrow niche of UCB-derived HSCs are the key factors for good outcomes of UCBT. Moreover, patient age and disease status at time of transplantation as described by the Pesaro classification including the extent of hepatomegaly >2 cm, portal fibrosis, and history of inadequate iron chelation therapy are limitations. The risks of graft failure of stem cell transplantation are hyperplastic bone marrow, allosensitization due to multiple transfusion, graft-versus-host-disease (GvHD), infections, and iron overload [28].

Gene therapy: To avoid allogenic hematopoietic stem cell transplantation and failure because of GvHD, gene therapy is a therapeutic option that harvests hematopoietic stem cells from the patient and corrects the target gene in vitro and then transplants the cells back to the patient in a personalized medicine approach. The delivery system is the key factor of this approach to increase the efficiency of targeting the genomic DNA. Moreover, this approach is limited by the type of mutation, there is no universal gene therapy for all types of mutation to cure all types of thalassemia [29]. The limitation of curative treatments, for both hematopoietic stem cell transplantation and gene therapy is a consequence of the high risk and high cost, as well as a lack of suitable facilities and specialized physicians.

Therefore, understanding the mechanism of ineffective erythropoiesis as the major cause of anemia in β-thalassemia and β-thalassemia/HbE diseases could lead to novel therapeutics and management strategies to improve the quality and duration of the lives of patients.

## 3. Molecular Mechanism of Ineffective Erythropoiesis in β-Thalassemia

The ineffective erythropoiesis in β-thalassemia is characterized by a massive increase in erythroblasts proliferation, coupled with increased erythroblast cell death resulting in low levels of circulating RBCs causing chronic anemia. Studies on the bone marrow of β-thalassemia patients have found increased numbers of early erythroid precursor cells, especially basophilic normoblasts and polychromatophilic normoblasts, with a decreased level of later-stage erythroblasts such as orthochromic normoblasts [30]. In addition, a changed myeloid to erythroid (M:E) cell ratio of 1:3 in β-thalassemia major as compared to the normal M:E ratio of 1.4:1 [30,31] has been observed. An erythrokinetic study in β^0^-thalassemia/HbE patients (six mild and five severe patients) measured RBC terminal half-life and erythrocyte iron utilization by intravenous injection of autologous RBC labeled with ^51^Cr, and the injection of ^59^Fe in the form of iron sulfate in donor plasma [3]. The severe β^0^-thalassemia/HbE patients had a shortened RBC survival in the peripheral blood circulation (9.2 ± 1.9 days) as compared to mild cases (17.0 ± 4.3 days), and both types of patients had shorted RBC survival times as compared to the reference range in normal subjects of 25.7 ± 0.9 days. Interestingly, while there was not a significant difference in erythrocyte iron utilization between severe (35.3 ± 8.9%) and mild cases (35.7 ± 9.0%), these levels were a significant decrease as compared to normal subjects (80%). The low iron utilization seen in β-thalassemia patients is consistent with the occurrence of a massive loss of developing erythroblasts. Though anemia in β-thalassemia is caused by both hemolysis and ineffective erythropoiesis, the erythrokinetic and ferrokinetic analyses indicated that ineffective erythropoiesis plays a much more prominent role than hemolysis. The significant question therefore is what happens to the differentiating erythroblasts in bone marrow of β-thalassemia patients that results in a lack of mature RBCs.

**Figure 3:**
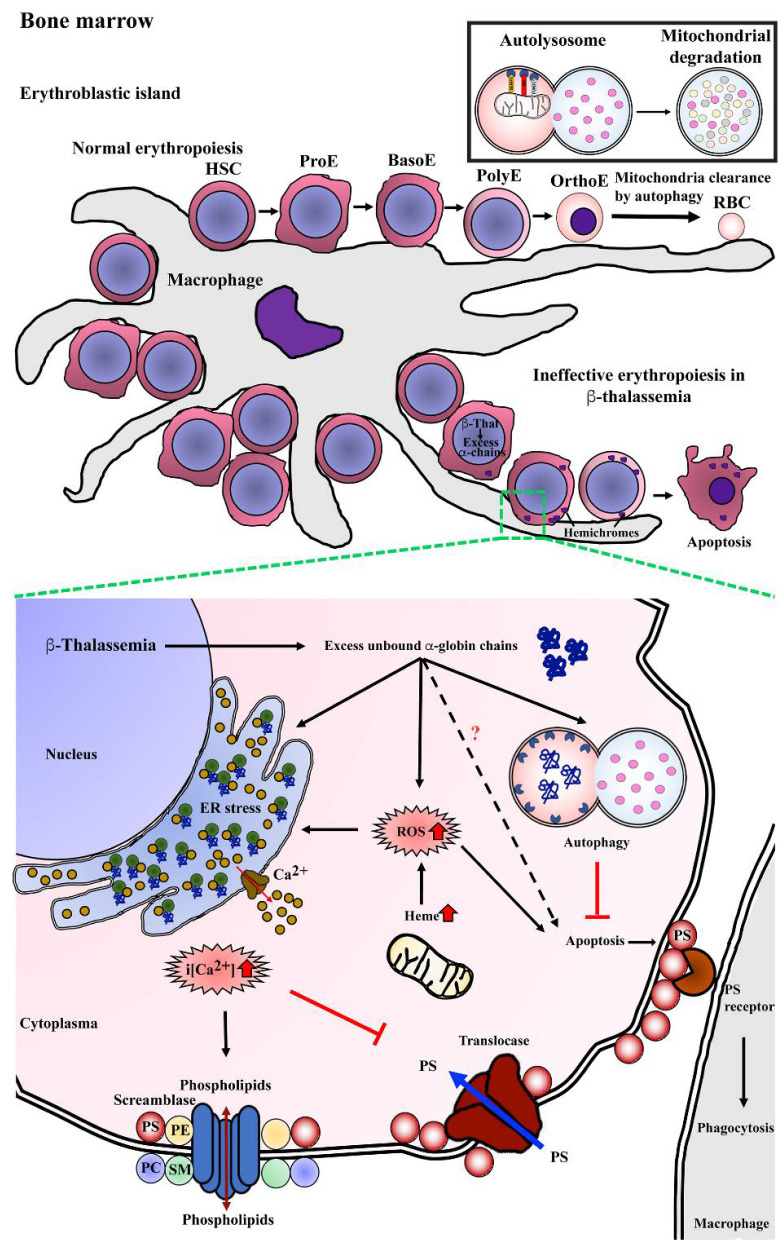
Mechanism of program cell death in bone marrow β-thalassemic erythroblasts. Normal erythropoiesis occurs in bone marrow in the location of an erythroblastic island that contains a macrophage as a feeder cell to product cytokines, which is essential for erythroid differentiation from hematopoietic stem cells (HSCs) to erythroid precursor cells including pronormoblasts (ProE), basophilic normoblasts (BasoE), polychromatophilic normoblasts (PolyE), and orthochromatic normoblasts (OrthoE) and consequently enucleation and mitochondria clearance by autophagy resulting in terminal erythroid differentiation and yielding mature red blood cells (RBCs). In β-thalassemia, excess unbound α-globin chains precipitate into erythroblasts leading to hemichrome accumulation and cellular stress. Autophagy could be a process of cellular adaptation in β-thalassemic erythroblasts to reduce the toxicity from excess unbound α-globin chains by protein degradation and inhibit apoptosis. However, the imbalance of α-globin/non-α-globin and increased heme in erythroblasts cause reactive oxygen species (ROS) generation via the Fenton reaction that consequently induces an ER stress response, resulting in the release of calcium (Ca^2^^+^) into cytoplasm. Increased intracellular calcium (i[Ca^2^^+^]) effects to activate scramblase but inhibits translocase, leading to the loss of plasma membrane asymmetry, resulting in increased phosphatidylserine (PS) on the outer membrane leaflet. PS-bearing erythroblasts could be cleared by macrophages via PS-PS receptor interaction as the “eat me” signal of phagocytosis.

Studies have shown that several factors contribute to ineffective erythropoiesis in β-thalassemia (Figure 3). Apoptosis has been proposed to be the cause of erythroblast death, and accelerated apoptosis of β-thalassemia erythroblasts has been demonstrated both ex vivo in the bone marrow and in vitro in erythroblast culture systems. DNA fragmentation (a hallmark of apoptosis) during the death of β-thalassemia major erythroblasts was shown by the presence of DNA ladders in DNA preparations from CD45^−^ bone marrow erythroblasts [31]. Similarly, bone marrow erythroblasts after culture for 7 and 14 days showed a majority of terminal deoxynucleotidyl transferase dUTP nick end labeling (TUNEL)^+^ basophilic normoblasts and polychromatophilic normoblasts [30]. Hoechst 33342^+^ erythroblasts have also been detected in cluster of differentiation (CD)45^−^ bone marrow cells of β-thalassemia patients [3]. In addition, increased phosphatidylserine (PS)-exposing basophilic normoblasts and polychromatophilic normoblasts have been reported in β-thalassemic bone marrow erythroblasts from both patients and from a mouse model [3,26,28].

Defects of β-globin chain synthesis in parallel with normal α-globin synthesis results in the presence of excess unbound α-globin chains. Accumulation and precipitation of excess α-globin chains in thalassemic erythroblast cytoplasm has been demonstrated in bone marrow erythroblasts obtained from β-thalassemia major and β-thalassemia/HbE as determined by electron microscope and laser confocal fluorescence microscopy [31,32]. The free heme released from the denatured excess α-globin chains could catalyze the formation of reactive oxygen species (ROS), such as hydroxyl radicals via the Fenton reaction, and consequently increase ROS in the thalassemic erythroblast. Bone marrow erythroblasts from the heterozygous β^IVS2−654^ thalassemic mouse model had a significantly increased α/β-globin ratio and increased ROS levels as compared to wild type mice, and there was a high correlation between the α/β-globin ratio and ROS levels at r = 0.92 [33]. Similar to the in vitro study, β^0^-thalassemia/HbE erythroblasts obtained from the culture of CD34^+^ peripheral blood stem cells showed increased ROS levels at day 7, 10, and 14 with basophilic normoblasts, polychromatophilic normoblasts, and orthochromic normoblasts being the majority erythroblast population on each day of culture examined, respectively [34]. The increased ROS can cause the progressive modification or degradation of cellular biomolecules, including DNA, protein, and lipids that could lead to loss of cell function and cell death.

The increased ROS generation is believed to contribute to increased lipid peroxidation, the loss of plasma membrane asymmetry and exposure of phosphatidylserine (PS) on the outer membrane lipid leaflet. In addition, excess unbound α-globin chains in β-thalassemic erythroblasts leads to ER stress. Oxidative stress together with ER stress could influence the flux of Ca^2+^ out of the ER, and as membrane lipid bilayer asymmetry is maintained by specific lipid transporters, such as amino-phospholipid translocase and phospholipid scramblase, increased Ca^2+^ can lead to membrane lipid asymmetry through lowered translocase activity and increased phospholipid scramblase activity consequently leading to PS exposure [35]. Increased scramblase activity in β-thalassemia mice erythroid cells has been previously demonstrated, and the majority of thalassemic erythroid cells with PS exposure have activated phospholipid scramblase [36]. Elevated levels of Ca^2+^ in β-thalassemia/HbE erythroblasts has been reported [37,38,39], and interestingly, reduction of Ca^2+^ in β-thalassemia/HbE erythroblasts by treatment with EGTA reduced the level of PS exposure to approximately normal control levels [39].

PS exposure on the cell surface is a signal for macrophages to phagocytose that cell, with clearance being needed as a consequence of senescence or damage. Transmission electron microscope analysis of bone marrow macrophages from β-thalassemia major patients showed intracytoplasmic inclusions consisting of phagocytosed erythroblasts and extruded erythroblasts at different stages of degradation [40]. Notably, a two-fold increase in macrophages with phagocytosed β-thalassemic bone marrow erythroblasts as compared to normal bone marrow erythroblasts has been reported [41]. The phagocytosis of β-thalassemic bone marrow erythroblasts was inhibited strongly by annexin V and PS-carrying vesicles, which confirms PS exposure as an “eat me” signal for macrophages [41]. Thus, increased PS exposure and perhaps other features of thalassemic erythroid precursors might lead to their enhanced phagocytic removal as another cause of ineffective erythropoiesis.

## 4. Autophagy

Autophagy is primarily a process in which the constituents of damaged or aged organelles are digested in double-membrane vesicles termed autophagosomes [42] with the constituents generated being available for further use in the cell. The term was originally coined by the Belgian cytologist and biochemist Christian De Duve, based on the ancient Greek word for “self-devouring”. Currently three main types of autophagy are recognized, macroautophagy (normally simply referred to as “autophagy”), microautophagy, and chaperone-mediated autophagy [43]. The mechanisms for autophagy, including the characterization of autophagy-related (Atg) genes in a yeast model were first elucidated by the cell biologist Yoshinori Ohsumi, who was awarded the 2016 Nobel Prize in Physiology or Medicine for his studies [44]. There are five key steps of the process of autophagy including initiation, vesicle nucleation, vesicle elongation, fusion, and degradation.

The pivotal complex in autophagy is the UNC1-like kinase 1 (ULK1) complex, consisting of ULK1, ATG13, FAK-family interacting protein of 200 kDa (FIP200) and ATG101. This ULK1 complex is largely regulated by two sensor molecules, mammalian target of rapamycin (mTOR) and AMP-activated protein kinase (AMPK) [45]. In the presence of nutrients, the mTOR complex 1 (mTORC1) interacts with the ULK1 complex through the regulatory-associated protein of mTOR (RAPTOR) leading to phosphorylation of ULK1 and ATG13, resulting in the inhibition of autophagy. Upon nutrient deprivation mTORC1 becomes no longer active, resulting in dephosphorylation of ULK1 and ATG13 and activation of ULK1 and autophagy induction [46]. Energy deprivation resulting in low ATP levels can also induce autophagy. Conditions of low cellular ATP (or an increase in the AMP: ATP ratio) results in the activation of the AMP-activated protein kinase (AMPK), which can remove the suppression of autophagy by phosphorylating the tuberous sclerosis complex 2 (TSC2), an mTORC1 inhibitor, by phosphorylating RAPTOR which inhibits mTORC1 activity, and by directly phosphorylating ULK1. Lastly, growth factor withdrawal can activate glycogen synthase kinase-3 (GSK3), which phosphorylates and activates acetyltransferase TIP60, which then acetylates ULK1 leading to its activation [47]. Collectively, these different stimuli result in activation of the ULK1 complex and the initiation of autophagy [48].

There are known to be a number of downstream targets of the ULK1 complex, including the complex itself, and ULK1 phosphorylates itself (autophosphorylation) as well as ATG13, ATG101, and FIP200 [49,50,51]. However, additional major targets are the Beclin 1 (BECN1)–class III phosphatidylinositol 3-kinase (PI3KC3) complexes whose activation results in the production of phosphatidylinositol 3-phosphate (PI3P), a critical signaling lipid required for the recruitment of autophagy effector molecules. The first complex, PI3KC3 complex 1 (PI3KC3-C1) consists of BECN1, ATG14L, Vps15, and Vps34 and mediates autophagosome initiation, while the second complex, PI3KC3-C2, consists of the same members except with the exchange of ATG14L for UVRAG, and is involved with autophagosome maturation.

Upon initiation of autophagy, the ULK1 mediated phosphorylation of BECN1, ATG14L, and Vps34 prompts formation of PI3KC3-C1 at the autophagosome biogenesis membrane site, believed to be part of the ER. The complex is anchored to the ER location through the action of activating molecule in BECN1 regulated autophagy (AMBRA), in a process that initiates phagosome biogenesis [52] at the phagophore assembly site (PAS). The PI3K produced by Vps34 prompts recruitment of DFCP1 and subsequently WIPI2b although their functions remain poorly elucidated [53,54,55]. The next Atg proteins recruited are those that are involved in two ubiquitylation-like reactions. In the first reaction Atg12 is conjugated to Atg5 in a process dependent upon Atg7 and Atg10, and the conjugates localize to the PAS, where they interact non-covalently with Atg16L. In the second conjugation reaction microtubule-associated protein 1 light chain 3 (MAP1-LC3; also known as Atg8 and LC3) is subjected to C-terminal cleavage by Atg4 forming cytosolic LC3-1, which is then covalently conjugated to the lipid phosphatidylethanolamine (PE) in a process requiring Atg7 and Atg3, generating LC3-II. The product of the first conjugation system (Atg5-Atg12-Atg16L) determines the site of LC3 lipidation, and thus LC3-II is specifically targeted to the expanding phagophores. Autophagosomes can fuse with endosomes to form amphisomes [56], and ultimately they fuse with lysosomes to form functionally complete autophagosomes (or autophagolysosomes) in which the vesicle content can be digested for recycling.

## 5. Autophagy during Normal Erythropoiesis

Erythropoiesis occurs at erythroblastic islands in the bone marrow that are characterized by developing erythroblasts surrounding a central macrophage (Figure 3). Macrophages act as nurse cells providing iron, cytokines, and chemokines for heme synthesis and erythroid development to generate mature RBCs from HSCs. The process of erythroid differentiation includes passage through a number of morphologically distinct stages from the earliest stage of pronormoblast, to basophilic normoblast, to polychromatophilic normoblast, and to orthochromic normoblast as the last stage before terminal erythroid differentiation (reticulocyte to mature RBC) (Figure 3). At terminal erythroid differentiation, orthochromic normoblasts expel their nuclei via an enucleation process and eliminate organelles such as mitochondria, the Golgi apparatus, the ER, and ribosomes, finally generating mature RBC [57,58]. Mitophagy is a subtype of autophagy that functions in mitochondrial clearance, and mitophagy can be considered as a type of targeted autophagy. As such, the mechanism of sequestration and degradation is largely identical to macroautophagy, and the significant differences lie in identification of the mitochondria as a target for degradation. Mitophagy is normally a cellular quality control mechanism in that the primary target is dysfunctional mitochondria [42]. However, mitophagy is also activated during remodeling of mitochondrial networks in a number of cell types [59,60,61] and in the elimination of mitochondria at terminal differentiation of erythroblasts [62]. While the molecular mechanisms of organelle clearance in erythropoiesis are not well understood, it is known that targeted deletion of autophagy genes, including *Ulk1* [63], *Fip200* [64], and *Atg7* [65] causes defective erythroid differentiation and anemia.

NIX-dependent removal of erythroblast mitochondria has been reported [62]. NIX, a BH3-only member of the Bcl-2 family, is localized on mitochondria and is up-regulated during terminal erythroid differentiation [4]. Nix has a specific role in targeting the mitochondria for clearance by mitophagy during erythroid maturation through the Nix-dependent loss of mitochondrial membrane potential (ΔΨm) and erythrocytes in the peripheral blood of *Ni*x^−/−^ mice exhibited mitochondrial retention [62]. *Nix*^−/−^ mice have normal autophagosome formation but multiple mitochondria outside of autophagosomes were observed. The deficiency in Nix inhibited the loss of ΔΨm during erythroid maturation and inducing the loss of ΔΨm with carbonyl cyanide p-trifluoromethoxyphenylhydrazone (FCCP), an uncoupling agent, promoted the clearance of mitochondria and restored the sequestration of mitochondria into autophagosomes in *Nix*^−/−^ reticulocytes.

A transcriptomic analysis of the *ATG* expression profile from human basophilic normoblasts showed increased expression of *ATG9A*, *ATG4A*, and *ATG3* but decreased expression of *ATG16L1*, *ATG13,* and *ATG12* when compared to undifferentiated HSCs and erythroid progenitor cells (HSPCs) [66]. There are four *ATG4* paralogs including *ATG4A*, *ATG4B*, *ATG4C,* and *ATG4D*. *ATG4B* is expressed throughout erythroid differentiation, while *ATG4A* and *ATG4D* levels were increased during terminal erythroid differentiation. Silencing of *ATG4A* expression in primary human CD34^+^ HSPCs using lentiviral shRNA targeting *ATG4A* resulted in delayed erythroid differentiation. In contrast, lentiviral shRNAs targeting *ATG4B* and *ATG4D* showed that these genes play a limited role in erythroid differentiation. A lentiviral vector expressing a fluorescent LC3B-ratiometric reporter (mCherry-EGFP-LC3B) was generated to investigate the LC3BI and LC3BII-PE formation in shATG4A transduced CD34^+^HSPCs. The depletion of ATG4A did not affect the conjugation of LC3B to PE, but reduced autophagic flux during erythropoiesis, and reduced clearance of mitochondria during terminal erythropoiesis were observed [66].

Atg7 plays a critical role in mitophagy. Atg7 regulates lipid conjugation of LC3-I to form lipid conjugated LC3-II. The role of Atg7 in regulating mitochondrial clearance in reticulocytes was demonstrated in *Atg7*^−/−^ mice, which presented with anemia and increased numbers of reticulocytes in blood circulation [67]. E14.5 fetal liver cells harvested from *Atg7*^−/−^ mice had no lipid-conjugated LC3-II and Atg5-Atg12 when compared to *Atg7*^+/+^ control mice. Differentiation of E14.5 fetal liver cells from *Atg7*^−/−^ mice to erythroblasts in a culture system showed reduced mitochondria and increased degradative vacuoles on day 1 as compared to day 0. However, unlike *Nix*^−/−^ mice, the percentage of mitochondria and degradative vacuoles in cells from *Atg7*^−/−^ mice was not significantly different when comparing day 0 and day 1. This finding implies that mitochondrial clearance in reticulocytes is regulated by both Atg7-dependent and -independent pathways.

Ulk1, a homolog of yeast Atg1, is critical for mitochondrial and ribosomal clearance during terminal erythroid differentiation, and the *Ulk1* expression pattern during erythropoiesis is similar to that of *Nix*. Peripheral blood samples of *Ulk1*^−/−^ mice showed mitochondrial and ribosomal retention in RBCs. The ΔΨm of *Ulk1*^−/−^ mice is well-maintained. Interestingly, treatment with carbonyl cyanide m-chloro phenyl hydrazone (CCCP), a potent mitochondrial uncoupling agent, leads to mitochondria clearance. These findings suggest that autophagy could be induced, and there are redundant Ulk1-independent mechanisms for mitochondria clearance [63].

## 6. Autophagy and Ineffective Erythropoiesis in Thalassemia

In β-thalassemia and β-thalassemia/HbE disease, erythroblasts have cellular stress as well as other pathological changes that could trigger autophagy (Figure 3), and as such during β-thalassemia erythropoiesis, autophagy plays roles other than simply being involved in mitophagy at terminal erythroid differentiation. An accumulation of misfolded proteins causes ER stress leading to an ER response via the activation of the unfolded protein response (UPR) pathway, which promotes cellular adaptation through several pro-survival mechanisms [68,69]. In normal cells, the UPR can respond to both internal and external stresses, which initiate detachment of the immunoglobulin heavy chain-binding protein (BiP, or glucose-regulated protein 78 (GRP78)) from three sensor proteins including activating transcription factor 6 (ATF6), inositol-requiring enzyme 1α (IRE1α), and PRKR-like ER kinase (PERK) [69,70]. Examination of the UPR response in erythroblasts from normal controls and β-thalassemia/HbE patients showed a significant lesion in the UPR response in erythroblasts from the patients [38]. While normal erythroblasts responded to both an internal stress (treatment with tunicamycin, an N-linked glycosylation inhibitor) and an external stress (removal of serum/cytokines) by activating the UPR, the patient-derived erythroblasts were insensitive to the external stress (serum removal), albeit that they responded appropriately to the internal stress (tunicamycin treatment). Collectively, this suggests that while the UPR in patient-derived erythroblasts is potentially active, there is a failure in transducing the external stimuli to the UPR. Studies have shown that levels of the cellular second messenger Ca^2+^ are increased in β-thalassemia mature erythrocytes [71] and pronormoblasts [37] as compared to normal control erythroblasts and erythrocytes, and interestingly reduction of cellular calcium levels in β-thalassemia/HbE erythroblasts was able to repair the transduction of the external stimuli to the UPR [38]. Prolonged ER stress can induce apoptosis [70], and serum deprivation induced apoptosis in both normal and patient-derived erythroblasts (as compared to non-serum deprived cells), but markedly the rate of apoptosis in the serum deprived patient cells was significantly higher than in the serum-deprived normal cells [70]. This indicates that β-thalassemia/HbE erythroblasts do not activate the UPR after serum withdrawal, but they are more affected by the results of the withdrawal than normal erythroblasts. Reduction of Ca^2+^ levels (which restored the UPR sensitivity to approximately normal levels) also reduced the level of apoptosis in response to serum withdrawal to approximately the same level as seen in serum withdrawal of normal erythroblasts [70].

Ca^2+^ has been implicated in the regulation of both the mTOR and AMPK pathways regulating autophagy [72], and increased autophagic flux has been observed in β-thalassemia/HbE erythroblasts as compared to cells derived from normal controls [39]. In a study undertaken in an erythroid culture system using CD34^+^ peripheral HSCs, increased autophagy was observed in patient-derived erythroblasts as compared to cells derived from normal controls. The time points observed (from day 7 through to day 13 of culture) would be before the onset of mitophagy, with cells consisting of basophilic normoblasts to orthochromic normoblasts [39]. As noted previously, erythroblasts from β-thalassemia patients have increased Ca^2+^ levels [37], and, as was seen with ER stress [38], modulation of Ca^2+^ levels significantly reduced levels of autophagy [39]. When autophagic flux was inhibited, normal cells showed increased levels of apoptosis, while patient-derived cells did not, and again modulation of Ca^2+^ levels increased levels of apoptosis to that seen in normal cells [39]. A more recent study examined the later stages of erythropoiesis in an essentially similar system [73], and observed that mitophagy was similarly increased in β-thalassemia erythroblasts as compared to cells derived from normal controls. It is also noteworthy that both studies [39,73] reported higher levels of apoptosis during differentiation in the normal control cells, with comparatively reduced levels in the β-thalassemia cells, suggesting that autophagy acts in a protective manner, offsetting the increased cellular stress resulting from imbalanced globin chain synthesis.

The protein quality control (PQC) system is critical for maintaining cellular protein homeostasis and integrity. Once protein homeostasis is impaired such as through misfolded proteins or protein aggregates that accumulate in the cell, the PQC can be triggered to detoxify and remove these damaged proteins. The PQC includes molecular chaperones, ubiquitin proteasome-dependent protein degradation and autophagy. The excess α-globin chain imbalance is the key factor leading to pathophysiological changes in β-thalassemic erythroblasts. Reduced toxicity from excess α-globin by being processed by ubiquitin-mediated proteolysis and autophagy has been demonstrated in β^th3/+^-thalassemia mice erythroblasts [74]. Inhibition of proteasome degradation using epoxomicin and MG132 led to increased insoluble α-globin accumulations in β^th3/+^-thalassemic mice erythroblasts. Moreover, the fluorescent proteasome activity indicator, MV151, showed increased proteasome activity in both nucleated and enucleated erythroid cells [74]. Autophagy also plays a role in α-globin degradation in β^th/+^-thalassemia mice erythroblasts, and inhibition of autophagic flux with chloroquine resulted in increased insoluble α-globin accumulations. Interestingly, inhibition of autophagic flux with chloroquine reduced α-globin degradation to a degree similar to that achieved through proteasome inhibition, while inhibition of both proteasome degradation and autophagy produced additive effects [74]. Autophagy degradation of excess insoluble α-globin in β^th/+^-thalassemia mice is mediated by ULK1, but independent of ATG5 [75]. Double mutant mice, β^th/+^ Ulk1^−/−^, have increased accumulation of insoluble α-globin in RBCs, and dense α-globin inclusions in reticulocytes, while only minor effects on insoluble α-globin accumulation were seen in β^th/+^Atg5^fl/fl^ double mutant mice. Systemic treatment of β^th3/+^-thalassemia mice with intraperitoneal rapamycin (an mTORC1 inhibitor) of 4 mg/kg/day for 30 days reduced α-globin precipitation in erythroblasts and improved pathology including RBC count, Hb levels, reticulocyte count, and size of spleen at twelve weeks after injection [75].

Apoptosis and autophagy are interconnected. Beclin1 acts as a key protein to initiate both autophagy and apoptosis, and the Beclin1/BCL2/BCL-X_L_ interaction inhibits the anti-apoptotic BCL family such as BCL2 and BCL-X_L_ but activates pro-apoptotic BCL family members such as BAX and BAK [76]. In contrast, the binding of Beclin1 and BCL2 is reduced when Beclin1 is phosphorylated by ULK1, AKT, and EGFR, and the initiation of vesicle nucleation of autophagy is activated [42,77]. Active caspase 3 is a critical protein in apoptosis, but additionally in autophagy as it cleaves ATG4D to promote autophagy during starvation, however, overexpression of caspase-mediated cleavage of ATG4D induced apoptosis. Moreover, there are reports that caspase-mediated cleavage of ATG5 and Beclin1 switches autophagy to apoptosis [78]. There are only a few studies about autophagy during erythropoiesis in β-thalassemia, and as such crosstalk between apoptosis and autophagy remains largely under investigated.

## 7. Novel Therapeutic Drugs Targeting Ineffective Erythropoiesis

Ineffective erythropoiesis is the main cause of pathology in β-thalassemia patients. Increased understanding of ineffective erythropoiesis has allowed the development of novel potential therapeutic options beyond the current standard of care. Here is an update on the novel therapeutic approaches that are currently in development at the clinical level (Table 1).

### 7.1. Janus-Associated Kinase (JAK) 2 Inhibitors

Erythropoietin, the master regulator of erythropoiesis, binds to the erythropoietin receptor to induce multiple signaling pathways that prevent apoptosis and support erythroid cell proliferation through the JAK2-STAT5 pathway [79]. In β-thalassemia, increased erythropoietin production secondary to chronic anemia and hypoxia consequently induce persistent phosphorylation of JAK2 causing massive erythropoiesis. Thalassemic mice treated with a JAK2 inhibitor showed improved erythropoiesis and reduced splenomegaly through a reduction of the excessive proliferation of early erythroblasts [80]. Ruxolitinib (INCB018424 or INC424) has been approved for the treatment of myelofibrosis and polycythemia vera by the US Food and Drug Administration (US-FDA) and the European Medicines Agency (EMA). A phase 2a single-arm multicenter clinical trial of ruxolitinib in thalassemia (NCT02049450), explored the efficacy and safety of ruxolitinib in 30 transfusion dependent β-thalassemia patients with spleen enlargement [81]. The patients received a starting dose of 10 mg ruxolitinib twice daily for 30 weeks. A reduction in spleen size from baseline was observed, but there was no improvement of pretreatment Hb levels or serum iron and ferritin levels. Thus, ruxolitinib has not proceeded to a phase 3 study.

### 7.2. Pyruvate Kinase Activator

As RBCs lack mitochondria required for ATP generation from the Krebs cycle and oxidative phosphorylation, ATP is largely generated by glycolysis. Pyruvate kinase is the last stage enzyme in glycolysis that convert phosphoenolpyruvate to pyruvate, generating ATP. In pyruvate kinase deficiency patients, an autosomal recessive disease, erythrocyte pyruvate kinase deficiency results in impaired glucose utilization and reduced ATP generation in RBCs, which leads to hemolysis. Mitapivat (AG-348) is an allosteric activator of RBC-pyruvate kinase. Oral administration of mitapivat in pyruvate kinase deficiency patients has been shown to increase ATP and Hb level with improvements in markers of hemolysis [82]. In a phase 2, open-label, multicenter clinical trial (NCT03692052), NTDT patients (5 α-thalassemia and 15 β-thalassemia) were administered mitapivat orally at 50 mg twice daily for the first 6 weeks, followed by an escalation to 100 mg twice daily for 18 weeks thereafter. The majority of the patients had increased Hb level from baseline [83]. Phase 3 studies are recruiting subjects for evaluating the efficacy and safety of mitapivat in TDT and NTDT of α-and β-thalassemia (NCT04770779 and NCT04770753, respectively).

### 7.3. Activin II Receptor Ligand Traps

Transforming growth factor-β (TGF-β) superfamily ligands play an essential role in the regulation of hematopoiesis, and these ligands are comprised of four protein groups, namely TGF-βs, activins, growth and differentiating factors (GDFs), and bone morphogenetic proteins (BMPs). TGF-β ligand receptors are composed of two types of serine and threonine kinase transmembrane receptors, the type I and type II receptors. Upon binding to ligands, the type II receptor phosphorylates and activates the type I receptor, and that consequently phosphorylates the R-Smad proteins, Smad2/3 for TGF-β and activin signaling, and Smad1/5/8 for BMP signaling [84]. Smad complexes are then translocated into the nucleus to regulate transcription of the target genes. Binding of ligands such as GDF11 to the activin type IIA receptor (ActRIIA) or the type IIB receptor (ActRIIB) causes inhibition of late-stage erythropoiesis and ineffective erythropoiesis [85,86]. Reduced binding of the ligands to the activin receptor II by activin receptor ligand traps leads to reduced SMAD2/3 signaling, consequently improving erythropoiesis by correction of maturation arrest and promoting terminal differentiation [85,86]. Two activin II receptor ligand traps, sotatercept (ACE-011) and luspatercept (ACE-536), have been evaluated in clinical trials and have shown promising results in β-thalassemia.

Sotatercept (ACE-011) is a dimeric recombinant fusion protein that consists of an extracellular domain of the human ActRIIA receptor linked to the Fc portion of human immunoglobulin G1 (IgG1). A phase 2a, open label, dose finding multicenter clinical trial in 16 TD β-thalassemia and 30 NTD β-thalassemia (NCT01571635) was performed [87]. Patients were treated with sotatercept through subcutaneous injection every 3 weeks at doses of 0.1, 0.3, 0.5, 0.75, or 1.0 mg/kg for ≤22 months to determine a safe and effective dose. The NTD β-thalassemia patients treated with 0.75–1.0 mg/kg sotatercept achieved sustained increases in Hb of ≥1.0 g/dL, while TD β-thalassemia patients treated with 0.75–1.0 mg/kg sotatercept achieved reductions of ≥33% in RBC transfusion requirements. While sotatercept is the first drug developed as an activin II receptor ligand trap to correct ineffective erythropoiesis, it binds to other members of the TGF-β superfamily such as activin A, and the development of luspatercept, a more selective activin II receptor ligand trap, obviated the need for further advanced trials [87].

Luspatercept (ACE-536) is a dimeric recombinant fusion protein that consists of the extracellular domain of the human ActRIIB receptor linked to the Fc portion of human IgG1. A phase 3, double-blind, randomized, placebo-controlled multicenter trial of luspatercept in TD β-thalassemia (BELIEVE; NCT02604433) has been undertaken [88]. A total of 332 β-thalassemia (224 patients in the luspatercept group and 112 in the placebo group) received luspatercept subcutaneously every 3 weeks at a dose of 1.00 to 1.25 mg/kg for at least 48 weeks. During any 12-week interval, the percentage of patients who had a reduction in transfusion burden of at least 33% was significantly greater in the treatment group than the placebo group, and as was the percentage of those having a reduction of at least 50%. In parallel, a phase 2, double-blind, randomized, placebo-controlled, multicenter study in 145 NTD β-thalassemia (BEYOND; NCT03342404) was undertaken [89]. Patients were randomly assigned to the luspatercept (*n* = 96) or placebo (*n* = 49) group, and for those in the treatment arm, luspatercept was given once subcutaneously every 3 weeks for 48 weeks starting at 1.0 mg/kg with escalation up to 1.25 mg/kg. The luspatercept group had an increase of at least 1.0 g/dL Hb concentration and a reduced transfusion burden. Luspatercept is the first disease-modifying drug for β-thalassemia, currently approved by the US-FDA in 2019 and the European Medicines Agency (EMA) in 2020 for TD β-thalassemia patients.

**Table 1:** Novel therapeutic drugs targeting ineffective erythropoiesis.

Drug	Mechanism of Action	Clinical Trial Identifier	Phase/Status *	Results	Reference
Ruxolitinib	Janus-associated kinase (JAK) inhibitors	NCT02049450	Phase 2 completed	No change in transfusion requirement, Reduction in spleen volume	[81]
Mitapivat (AG-348)	Pyruvate kinase activator	NCT03692052	Phase 2 completed	Increase Hb concentration	[83]
Mitapivat (AG-348)	Pyruvate kinase activator	NCT04770779, NCT04770753	Phase 3 recruiting		
Sotatercept (ACE-011)	Activin II receptor ligand traps	NCT01571635	Phase 2 completed	Increased Hb concentration, Reduced transfusion burden	[87]
Luspatercept (ACE-536)	Activin II receptor ligand traps	NCT03342404	Phase 2 active, not recruiting	Improves Hb concentration, Reduces transfusion burden	[89]
Luspatercept (ACE-536)	Activin II receptor ligand traps	NCT02604433	Phase 3 completed	Reduces transfusion burden	[88]

* From ClinicalTrials.gov. (accessed on 2 August 2022) Hb; hemoglobin.

## 8. Conclusions and Future Perspective

β-Thalassemia, one of the most common genetic inheritance disorders worldwide, is caused by the absence or reduced presence of β-globin chains in Hb molecules [1]. It is distributed worldwide, and can be found in the Mediterranean region, the Middle East, the Indian subcontinent, and throughout Southeast Asia. However, increased global migration is a major factor that has increased the prevalence of β-thalassemia worldwide, leading to it becoming a global, rather than regional public health problem. Sadly, many of the affected populations do not have adequate access to appropriate management and treatment, leading to tens of thousands of deaths each year. Even where treatment is available, the requirement for regular transfusions leads to iron overload in patients and consequent systemic problems. Iron chelation therapy can relieve some of the overloading, but these patients still have increased morbidity and mortality as compared to those without thalassemia. In many ways, current treatments for thalassemia have been developed to treat specific symptoms—splenectomy to remove enlarged spleens, blood transfusions to treat anemia, and then chelation therapy to treat the consequent iron overloading. The last decade has seen the introduction of curative treatments such as stem cell transplantation and gene therapy. While these provide long-term cures, their cost and requirement for specialized facilities and physicians put them out of the reach of the vast majority of patients. A number of new drugs are in current trials, and these stem from our increased understanding of the process of erythropoiesis and how it becomes dysregulated in cases of β-thalassemia disease.

At its core, ineffective erythropoiesis is the main cause of anemia and the consequent pathology in β-thalassemia, and it is caused by excess unbound α-globin precipitation in erythroblasts, leading to cell death. However, there are still several questions about the molecular mechanisms leading to the death of β-thalassemic erythroblasts. This is in part because cell culture systems do not fully recapitulate the massive cell death at the polychromatophilic normoblast stage, and it is critical to future advancement in this field that this shortcoming in our current technology is overcome. However, there are several reports that β-thalassemic erythroblasts die in the bone marrow via apoptosis as demonstrated by the presence of a classic DNA ladder, as well as TUNEL and annexin V positive signals [3,26,27], but as yet the detailed mechanism of apoptosis from both the intrinsic and extrinsic pathways has not been elucidated. Intercellular communication is also important. The interaction of cells in the bone marrow microenvironment including the erythroblastic islands needs further investigation, possibly through the development of co-culture systems that more faithfully recreate the bone marrow microenvironment. This might shed light on extrinsic signals leading to cell death and the process of removal by macrophages.

Increased autophagy in β-thalassemic erythroblast is well-documented [35,70,71]. However, if the basal prosurvival mechanism of autophagy becomes overwhelmed by the constant stress imposed by the unpaired α-globin chains, it is possible that autophagy may promote cell death. Any cross-talk in β-thalassemic erythroblasts between apoptosis and autophagy remains largely unclear, and how any interplay between these two mechanisms contribute to ineffective erythropoiesis requires more detailed investigation. Autophagy has been shown to reduce excess α-globin precipitations in erythroblasts. Stimulating autophagic clearance of excess α-globin could be a potential therapeutic option for β-thalassemia as inhibition of mTORC1 showed reduced α-globin precipitation in erythroblasts and improved hematological parameters in β^th3/+^-thalassemia mice [75]. However, a lot more work needs to be done to prove the concept and develop the prospect of a new treatment approach.

## Data Availability

Not applicable.
